# Supplementary material for: SCMFMDA: Predicting microRNA-disease associations based on similarity constrained matrix factorization
Source: PLoS Comput Biol. 2021 Jul 12;17(7):e1009165. doi: 10.1371/journal.pcbi.1009165 (PMC8345837; doi:10.1371/journal.pcbi.1009165)
Supplement: Attachment — Submitted filename: Summary of changes .docx [file pcbi.1009165.s008.docx]

The Author's Response to the Reviewer's Comment on PCOMPBIOL-D-21-00629

**“SCMFMDA: Predicting microRNA-disease Associations based on Similarity Constrained Matrix Factorization”**

Lei Li, Zhen Gao, Yu-Tian Wang, Ming-Wen Zhang, Jian-Cheng Ni*, and Chun-Hou Zheng*, *Member, IEEE*

We sincerely thank the editor and the reviewers for their constructive comments and suggestions concerning our manuscript entitled “SCMFMDA: Predicting microRNA-disease Associations based on Similarity Constrained Matrix Factorization” (ID: PCOMPBIOL-D-21-00629). We have carefully revised the manuscript according to these constructive comments, and my revised paper must include the abstract, index terms, author affiliation information, main text, references, figure captions, table titles, brief author biographies. The responds to the reviewer’s comments are listed as follows.

**Reviewer #1**: **In this study, Li et al. proposed a computational model to predict miRNA-disease associations based on similarity constrained matrix factorization. Their method, SCMFMDA, achieved better performance than existing ones and accurately predict relations between miRNAs and diseases in the following case studies. The model seems valid and the results they show are promising. However, following concerns should be addressed before considering for publication.
There are three major issues for model validation:**

**First, the authors should provide more details about the dataset they used for validation. They only mentioned: “Based on the verified association of miRNA-disease in HMDD V2.0 database”, which is not enough for the readers to fully understand the dataset. For examples, how many verified associations included in the dataset? How many unknown cases in the dataset? Do they perform any filtering to the dataset?**

**Response**: Thanks for your comments. We have specifically introduced the HMDD v2.0 database in Materials, which only included verified 5430 associations between 383 diseases and 495 miRNAs. In addition to 5430 verified associations, the other 184155 associations belong to unknown cases. And we don’t perform any filtering to the dataset. We have provided more details about the dataset that are used in model validation.

“Based on the verified associations of miRNA-disease in HMDD v2.0 database, global Leave-one-out cross validation (global LOOCV) and five-fold cross validation (5-CV) were implemented to evaluate the prediction performance.”

“Based on the information of HMDD v2.0 database that included 5430 verified associations and 184155 unverified associations between 383 diseases and 495 miRNAs, global Leave-one-out cross validation (global LOOCV) and five-fold cross validation (5-CV) were implemented to evaluate the prediction performance. In the framework of global LOOCV, the test set was held by each verified association of miRNA-disease in turn, the training set was composed of other verified associations. The whole unknown miRNA-disease associations were considered as candidate samples. Similarly, in the framework of 5-CV, the whole verified miRNA-disease associations were divided into five parts in a random way, where test set was held by one part in turn, training set consisted of other four parts in turn. The whole unknown miRNA-disease associations were considered as candidate samples.”

**Second, the authors should summarize the existing methods they compete and show the information including the publication time, the mathematic model they use, the dataset they used for training etc. in a table. The authors should also provide the details about how they compare these methods. To my understanding, it’s better to compare different methods in an independent testing set instead of on their own training set. Different methods can be trained on different datasets, it’s unclear how the authors compare these methods using cross validation.**

**Response**: Thanks for your comments. We have summarized the existing methods and show the specific information of these methods. In order to make the comparison results become fairness, these methods all used HMDD v2.0 database to train. The HMDD v2.0 database included 5430 verified associations and 184155 unverified associations between 383 diseases and 495 miRNAs. In the framework of global LOOCV, the test set was held by each verified association of miRNA-disease in turn, the training set was composed of other verified associations. The whole unknown miRNA-disease associations were considered as candidate samples. Similarly, in the framework of 5-CV, the whole verified miRNA-disease associations were divided into five parts in a random way, where test set was held by one part in turn, training set consisted of other four parts in turn. The whole unknown miRNA-disease associations were considered as candidate samples. After all datasets were divided, by either the global LOOCV method or the 5-CV method, these models are carried out to obtain the predicted score for all associations, respectively. The ranks of the test samples in relative to those of the candidate samples are then calculated. Subsequently, the receiver operating characteristics (ROC) curves at different thresholds are plotted using the false positive rate (FPR, 1-specificity) as the abscissa and the true positive rate (TPR, sensitivity) as the ordinate. Finally, the areas under the ROC curve (AUC) are determined: the higher the AUC value, the better the prediction performance of the model.

In order to evaluate the prediction ability of SCMFMDA, we compared several previous computational methods that were proposed to predict unknown miRNA-disease associations. We applied same dataset (HMDD v2.0 database) to train these methods so that comparison results could be considered as fairness. The specific information of these methods are shown as follows.

- MSCHLMDA [55] is a multi-similarity based combinative hypergraph learning model (published in 2020).
- ICFMDA [56] is an improved collaborative filtering-based computational model (published in 2018).
- SACMDA [57] is short acyclic connections-based computational model (published in 2018).
- GRNMF [58] is a graph regularized non-negative matrix factorization-based model (published in 2018).
- ${GRL}_{2,1}-NMF$ [59] is a graph Laplacian regularized $L_{2,1}$-nonnegative matrix factorization-based computational model (published in 2020).
- NPCMF [60] is a nearest profile-based collaborative matrix factorization model (published in 2019).
- KBMFMDA [61] is a kernelized Bayesian matrix factorization-based computational model (published in 2020).

Based on the HMDD v2.0 database that included 5430 verified associations and 184155 unverified associations between 383 diseases and 495 miRNAs, global Leave-one-out cross validation (global LOOCV) and five-fold cross validation (5-CV) were implemented to evaluate the prediction performance of these methods. In the framework of global LOOCV, the test set was held by each verified association of miRNA-disease in turn, the training set was composed of other verified associations. The whole unknown miRNA-disease associations were considered as candidate samples. Similarly, in the framework of 5-CV, the whole verified miRNA-disease associations were divided into five parts in a random way, where test set was held by one part in turn, training set consisted of other four parts in turn. The whole unknown miRNA-disease associations were considered as candidate samples. In addition, by either the global LOOCV or the 5-CV, we applied SCMFMDA to obtain all predicted association scores so that the ranking of test set relative to candidate samples could be calculated. When the ranking of all test sample were higher than the certain threshold, SCMFMDA was regarded as a valid model. Then we could utilize the Receiver operating characteristics (ROC) curve that was obtained by plotting the true positive rate (TPR) against the false positive rate (FPR) to effectively evaluate the performance of SCMFMDA. We could calculate the area under the ROC curve (AUC) of SCMFMDA whose value was between 0 and 1. Similarly, we could obtain AUCs of other computational methods by utilizing the information of HMDD v2.0 database.

In this work, when global LOOCV method was conducted, SCMFMDA, MSCHLMDA, ICFMDA and SACMDA acquired average AUC values of 0.9675, 0.9287, 0.9072 and 0.8777, respectively (Fig. 4). For the purpose of reducing potential deviations resulted in random sample segmentations, we applied 100 times repeated segmentations to verified associations of miRNA-disease in 5-CV method, and the average AUC values of SCMFMDA, MSCHLMDA, ICFMDA and SACMDA reached 0.9447, 0.9263, 0.9046, and 0.8773, respectively (Fig. 5). Obviously, the prediction performance of SCMFMDA was better than other methods.

**Third, the authors should provide the dataset they use as well as the source codes to execute their and others’ models, so that others can reproduce their results to confirm the correctness.**

**Response**: Thanks for your comments. We will provide the dataset and source codes, so that the results can be reproduced to confirm the correctness.

**As for parameter optimization, I’m not sure why they first fix two to optimize the third and then fix the third to optimize other two. And they don’t mention the exact values of two parameters they use to optimize the third. Just randomly pick some values? A more straightforward way is to test all the 490 possible combinations of three parameters and select the combination with best performance. Is this strategy very time-consuming? Figure 5a is not very professional, the dots representing the exact values should be plotted on the figure with a more smoothed curve to show the trend. The first two values on the figure is weird, why there is no value for 0 but two values for 10%? A color bar should be added for the heatmap in figure 5b. They grey line surround the heatmap can be removed.**
**Response**: Thanks for your comments. We have explained why we first fix $\sigma$ and $\varepsilon$ to optimize the $\gamma$ and then fix the $\gamma$ to optimize $\sigma$ and $\varepsilon$. And we have re-modified the figures in the revised manuscript.

(a)

(b)

Fig.3. The influence of parameters on SCMFMDA. (a) the influence of $\gamma$ (b) the influence of $\sigma$ and $\varepsilon$.

The AUC value of 5-CV is applied to evaluate influence of the choice of parameters on the performance of model. And after generous test experiments were conducted, we could get the conclusion that the value of $\gamma$ would affect the experiment individually. For this reason, we fixed $\sigma$ and $\varepsilon$ in a suitable combination to test the most suitable value of $\gamma\in\{0, 10\%,\ldots1\}$ in SCMFMDA. In order to ensure the correctness of the test, $\sigma$ and $\varepsilon$ are fixed in different combination. From Figure 3a, we could see that SCMFMDA obtained the best performance when$\gamma=50\%$. In addition, the $\gamma=50\%$ is fixed so that the effect of regularization parameters $\sigma$ and $\varepsilon$ can be clearly evaluated. We utilized all combinations of $\sigma\in\left\{ 2^{-3},2^{-2},\ldots,2^{3} \right\}$ and $\varepsilon\in\left\{ 2^{-3},2^{-2},\ldots,2^{3} \right\}$ to construct SCMFMDA. From Figure 3b, we could discover that SCMFMDA acquired best AUC value of 0.9447 when $\sigma=2^{2}$ and $\varepsilon=2^{0}$.
**For case studies, a more detailed statistics of the performance can be shown in addition to three tables. For examples, for each disease, how many percentages of miRNAs in the top 50 can find evidences in HMDD v3.2 or dbDEMC v2.0 or both? How many cannot find clues in the database. Also, what’ s the differences between HMDD v3.2 and v2.0, which is used for model training? Are there any overlaps? if so, the authors should exclude those involved in the model training.
Response**: Thanks for your comments. We have added more detailed statistics of the performance. And we use “HMDD v3.2 database” to instead of “miR2Disease database” so that prediction performance can be effectively validated.

For a specific disease, verified associations of whole diseases in HMDD v2.0 database are considered as training samples, unverified associations with the specific disease in HMDD v2.0 database are treated a candidate samples. By training this model, we could rank predicted association score of the candidate samples and then the top 50 candidate associations with the specific disease are selected. In addition, we utilized two types of databases that were miR2disease and dbDEMC v2.0 to check out miRNAs that have been ranked. Moreover, Table II and Table III indicated prediction results obtained via SCMFMDA, respectively. The 94% and 92% of top 50 miRNAs that inferred by our model, which were individually confirmed to associate with colon neoplasms and lung neoplasms according to the miR2Disease and dbDEMC v2.0 databases. Only 3 and 4 of top 50 predicted miRNAs that are related colon neoplasm and lung neoplasms could not find clues in the databases.

[42] Q. Jiang, Y. Wang, Y. Hao, J. Liran, M. Teng, X. Zhang, M. Li, G. Wang, Y. Liu, “miR2Disease: a manually curated database for microRNA deregulation in human disease,” *Nucleic Acids Research*, vol. 37, no. 1, pp. D98-D104, 2009.

**The organization of the “Results” part is not quite reasonable. Logically, the parameter optimization should be in front of model comparison. Model comparison can be separated to non-MF methods and MF methods, but I would suggest putting them together. The last part can be the case studies to further confirm the prediction value of the method. In this way, the reader can better follow the logic of the results.**

**Response:** Thanks for your comments. In order to make the readers can better follow the logic of the results, we have made appropriate adjustments to make the “Results” part become reasonable. Now, the “Result” part sequentially includes “Parameters optimization”, “Model comparison”, and “Case studies”. And we put non-MF methods and MF methods comparison in “Model comparison” together.

**Reviewer #2**: This paper proposed a new approach for miRNA-disease associations prediction. Experimental results indicated that this approach can effectively and efficiently predict miRNA-disease associations. This manuscript is well-written, which makes it easy to understand, although there are some minor issues to be addressed. I have the following specific comments.

**1. There are some typos and grammatical errors throughout the paper, the authors should recheck the whole paper carefully to revise these problems.**

**Response**: Thanks for your comments. The paper has been carefully revised to correct these typos and grammatical errors.

**2. There is an error in Equation (26) and Equation (32) in section of METHODS (D. Optimization algorithm). The** $\boldsymbol{A(:,j)}$ **should be changed to** $\boldsymbol{A(:,j)}^{\boldsymbol{T}}$**. The authors should revise this error.**

**Response**: Thanks for your comments. We have corrected this mistake of Equation (26) and Equation (32) as follows:

$$\begin{aligned} \nabla_{v_{j}}L=\sum_{j} \left( v_{j}u_{i}^{T}-a_{ij} \right)u_{i}+\sigma v_{j} \\ +\varepsilon\left( \sum_{i} \left( v_{j}-v_{i} \right)S_{ji}^{m}-\sum_{i} \left( v_{i}-v_{j} \right)S_{ij}^{m} \right) \\ =v_{j}\left( U^{T}U+\sigma I+\varepsilon\left( \sum_{i} S_{ij}^{m}+\sum_{i} S_{ji}^{m} \right)I \right) \\ -{A\left( :,j \right)}^{T}U-\varepsilon\sum_{i} (S_{ij}^{m}+S_{ji}^{m})v_{i}\#\left( 26 \right) \end{aligned}$$

$$\begin{aligned} v_{j}\leftarrow\left( {A\left( :,j \right)}^{T}U-\varepsilon\sum_{i} (S_{ij}^{m}+S_{ji}^{m})v_{i} \right) \\ \left( U^{T}U+\sigma I+\varepsilon\left( \sum_{i} S_{ij}^{m}+\sum_{i} S_{ji}^{m} \right)I \right)^{-1}\#\left( 32 \right) \end{aligned}$$

**3. The authors ranked the miRNAs associated with diseases in section of RESULTS (C. Case studies). In my opinion, the authors should specifically introduce how to get the ranking of these miRNAs.**

**Response**: Thanks for your comments. We have specifically introduced how to get the ranking of these miRNAs in this revised manuscript.

“For a specific disease, verified associations of whole diseases in HMDD v2.0 database are considered as training samples, unverified associations with the specific disease in HMDD v2.0 database are treated a candidate samples. By training this model, we could rank predicted association score of the candidate samples and then the top 50 candidate associations with the specific disease are selected.”

**4. Authors compared this model with other MF-based models in section of RESULTS (D. Comparison with MF-based Models). Many matrix factorization-based models have developed for miRNA-disease prediction in a recent time. I think you should compare your model with them.**

**Response**: Thanks for your comments. We have compared our model with other MF-based models that published in a recent time in this revised paper.

“In order to reflect the performance of the SCMFMDA, it is compared with other previous matrix factorization-based models that include GRNMF [61], IMIPMF [62], SNMFMDA [63], KBMF-MDI [64].”

**Table I. Comparisons between SCMFMDA and other MF-based models**

| Computational models | AUC of 5-CV |
| --- | --- |
| GRNMF | 0.869 |
| IMIPMF  SNMFMDA  KBMF-MDI  SCMFMDA | 0.891  0.8426  0.8815  0.9447 |

“In order to reflect the performance of the SCMFMDA, it is compared with other state-of-the-art matrix factorization-based models that include GRNMF, ${GRL}_{2,1}-NMF$, NPCMF, KBMFMDA.”

**Table I. Comparisons between SCMFMDA and other MF-based models**

| Computational models | AUC of 5-CV |
| --- | --- |
| GRNMF | 0.869 |
| ${GRL}_{2,1}-NMF$  NPCMF  KBMFMDA  SCMFMDA | 0.9276  0.9429  0.9008  0.9447 |

[58] Q. Xiao, J. Luo, C. Liang, J. Cai, and P. Ding, “A graph regularized non-negative matrix factorization method for identifying microRNA-disease associations,” *Bioinformatics*, vol. 34, no. 2, pp. 239-248, Jan. 2018.
[59] Z. Gao, Y. Wang, Q. Wu, J. Ni, and C. Zheng, “Graph regularized $L_{2,1}$-nonnegative matrix factorization for miRNA-disease association prediction,” *BMC Bioinformatics*, vol. 21, p. 61, Feb. 2020.
[60] Y. Gao, Z. Cui, J. Liu, J. Wang, C. Zheng, “NPCMF: Nearest Profile-based Collaborative Matrix Factorization method for predicting miRNA-disease associations,” *BMC Bioinformatics*, vol. 20, no. 1, p. 353, 2019.
[61] X. Chen, S. Li, J. Yin, C. Wang, “Potential miRNA-disease association prediction based on kernelized Bayesian matrix factorization,” *Genomics*, vol. 112, no. 1, pp. 809-819, Jan. 2020.

**5. In your paper, the “Gaussian interaction profile kernel similarity” are referred to as “GIP kernel similarity”, but you used “GIP similarity” to referred in a few places in your paper. The authors should pay attention and make changes.**

**Response**: Thanks for your comments. We have carefully check this question in our paper and change all “GIP similarity” to “GIP kernel similarity” in this revised manuscript.

**6.** **Many important computational models for miRNA-disease association prediction published in the top journals such as Bioinformatics and IEEE/ACM Transactions on Computational Biology and Bioinformatics should be discussed and cited. This research field has made much progress in recent several years. Author hardly cite recent computational studies for example:**

**Y. Zhao, X. Chen, and J. Yin, “Adaptive boosting-based computational model for predicting potential miRNA-disease associations,” *Bioinformatics,* vol. 35, no. 22, pp. 4730-4738, Apr. 2019.**

**C. Ji, Y. Wang, Z. Gao and L. Li, “A Semi-Supervised Learning Method for MiRNA-Disease Association Prediction Based on Variational Autoencoder,” vol.1, no. 1, p.99, Mar. 2021.**

**Response:** Thanks for your comments. We have cited these recent computational studies.

Zhao et al. [23] applied adaptive boosting to observe unverified miRNA-disease association in ABMDA model. And they utilized k-means clustering on negative samples to perform random sampling, which could control the balance between positive samples and negative samples.

Ji et al. [40] proposed SVAEMDA model to infer more disease-related miRNAs, which used miRNA similarity and disease similarity to obtain the representations of miRNA and disease. In addition, the variational autoencoder based predictor was trained to predict unknown interactions of miRNA-disease, which combined verified miRNA-disease interactions with the representations of miRNA and disease to generate the feature vectors of miRNA and disease.

[23] Y. Zhao, X. Chen, and J. Yin, “Adaptive boosting-based computational model for predicting potential miRNA-disease associations,” *Bioinformatics*, vol. 35, no. 22, pp. 4730-4738, Apr. 2019.
[40] C. Ji, Y. Wang, Z. Gao and L. Li, “A Semi-Supervised Learning Method for MiRNA-Disease Association Prediction Based on Variational Autoencoder,” *IEEE/ACM Trans Computational Biology & Bioinformatics*, vol.1, no. 1, p.99, Mar. 2021.

**Reviewer #3**: In this paper, the authors use similarity network fusion and similarity constrained matrix factorization for miRNA-disease association prediction (SCMFMDA), which completes the missing associated scores between miRNAs and diseases. According to the relevant results, it demonstrates that SCMFMDA is effective for prediction of possible associations of miRNA-disease. However, there are existing some detailed problems.
**1. Authors should revise your English writing carefully and eliminate small errors in the paper to make the paper easier to understand.**

**Response**: Thanks for your comments. We have carefully revised the English writing and eliminate some small errors in our paper.

**2. Authors should explain in details the function and impact of parameter ε in Equation (24).**

**Response**: Thanks for your comments. We have explained in details the function and impact of parameter ε in Equation (24).

“where hyper parameter $\varepsilon$ controls the similarity consistency.”

“where $\varepsilon$ is regarded as hyper parameter which can availably control the smoothness of similarity consistency.”

**3. The AUC values of global LOOCV of SCMFMDA, MSCHLMDA, GRL-NMF, IMCMDA, ICFMDA and SACMDA were introduced in the section of RESULTS (A. Model validation), but the corresponding values in Fig. 3 look different.**

**Response**: Thanks for your comments. We have corrected it in this revised manuscript.

“Figure 3 indicated that SCMFMDA, MSCHLMDA, ${GRL}_{2,1}-NMF$, IMCMDA, ICFMDA and SACMDA acquired AUC values of 0.9675, 0.8777, 0.8384, 0.9287, 0.9072 and 0.9280, respectively.”

“In this work, when global LOOCV method was conducted, SCMFMDA, MSCHLMDA, ICFMDA and SACMDA acquired average AUC values of 0.9675, 0.9287, 0.9072 and 0.8777, respectively (Fig. 4).”

**4. There is a reference error in Table III. Authors use ‘a’ and ‘b’ to denote ‘HMDD v3.2 database’ and ‘dbDEMC v2.0 database’ in Table III, respectively, but label ‘H’ and ‘d’ to denote ‘HMDD v3.2 database’ and ‘dbDEMC v2.0 database’ under the Table III.**

**Response**: Thanks for your comments. We have used “miR2Disease database” instead of “HMDD v3.2 database” and apply ‘a’ and ‘b’ to indicate ‘miR2Disease database’ and ‘dbDEMC v2.0 database’, respectively.

**5. Authors should pay attention to formatting errors which should be further adjusted. For example, there are some fonts are so small in Fig. 2.**

**Response**: Thanks for your comments. We have carefully revised these formatting errors. And we have adjusted the size of fonts in Fig. 1 and Fig. 2.
