# Supplementary material for: SCMFMDA: Predicting microRNA-disease associations based on similarity constrained matrix factorization
Source: PLoS Comput Biol. 2021 Jul 12;17(7):e1009165. doi: 10.1371/journal.pcbi.1009165 (PMC8345837; doi:10.1371/journal.pcbi.1009165)
Supplement: Attachment — Submitted filename: Summary of changes.docx [file pcbi.1009165.s009.docx]

The Author's Response to the Reviewer's Comment on PCOMPBIOL-D-21-00629R1

**“SCMFMDA: Predicting microRNA-disease Associations based on Similarity Constrained Matrix Factorization”**

Lei Li, Zhen Gao, Yu-Tian Wang, Ming-Wen Zhang, Jian-Cheng Ni*, and Chun-Hou Zheng*, *Member, IEEE*

We sincerely thank the editor and the reviewers for their constructive comments and suggestions concerning our manuscript entitled “SCMFMDA: Predicting microRNA-disease Associations based on Similarity Constrained Matrix Factorization” (ID: PCOMPBIOL-D-21-00629R1). We have carefully revised the manuscript according to these constructive comments, and my revised paper must include the abstract, index terms, author affiliation information, main text, references, figure captions, table titles, brief author biographies. The responds to the reviewer’s comments are listed as follows.

**Reviewer #1**: **The authors have made substantial efforts to address the reviewers' concerns and the revised manuscript improved from many aspects. I have one more question, in the data availability section, the authors claimed "All relevant data are within the manuscript and its Supporting Information files", yet I haven't found the code and datasets they used in their supporting information files or any link to public website for data sharing in their manuscript. Do I miss it or they don't provide it?**

**Response**: Thanks for your comments. We have placed the code and datasets we used in the Supporting Information files.

**Reviewer #2**: **All my concerns have been solved.**

**Reviewer #3**: **The question of the manuscript has been answered correctly.**
